# Navigating dual roles: qualitative exploration of the psychological impacts on Muslim professionals supporting their community after a terror attack

**DOI:** 10.1192/bjo.2025.10897

**Published:** 2025-11-05

**Authors:** Ruqayya Sulaiman-Hill, Fareeha Ali, Rana Lotfy Ahmed, S. M. Akramul Kabir

**Affiliations:** Department of Psychological Medicine, University of Otago, Christchurch, New Zealand

**Keywords:** Terrorism, dual relationships, psychosocial impacts, community support, trauma- and stressor-related disorders

## Abstract

**Background:**

Professionals engaged in community work within their own communities frequently encounter challenges associated with dual relationships. The psychological impacts of dual roles are often overlooked.

**Aims:**

This study explores the experiences of Muslim professionals in Christchurch, New Zealand, following the 15 March 2019 mosque terrorist attacks. It examines how they balance their community roles with their professional responsibilities while also safeguarding their personal well-being.

**Method:**

Semi-structured interviews were conducted with 20 Muslim professionals engaged in dual relationships within their community. Participants were selected through purposive sampling from diverse sectors, including government agencies, research positions and community support services. Reflexive thematic analysis was utilised to identify key themes.

**Results:**

Participants reported significant emotional strain, including vicarious trauma and burnout, driven by their dual roles. Faith emerged as a key motivator, with altruism framed as a spiritual duty. Identity struggles were common, shaped by societal scrutiny and a desire for validation. While formal support systems were sometimes inadequate, peer support and culturally attuned leadership provided relief. The findings highlight the complex interplay of psychological, spiritual and structural factors in sustaining professionals following a disaster.

**Conclusions:**

This research highlights the emotional toll on Muslim professionals supporting their community following a terrorist attack, with broader implications for minority groups responding to similar incidents. The findings highlight the need for culturally competent, trauma-informed support systems within community care organisations. Recommendations include strengthening of peer support, training supervisors in cultural responsiveness and ensuring tailored mental health resources to support well-being and professional effectiveness in high-impact roles.

On 15 March 2019, a terrorist motivated by White supremacist ideology carried out an attack on two mosques in Christchurch, New Zealand during Friday congregational prayers. This resulted in the loss of 51 lives and left numerous individuals injured, marking it as one of the most severe mass shootings in New Zealand’s recorded history.^1^ The impact resonated deeply within the approximately 4000-member Muslim community in Christchurch, which needed to confront not only the immediate aftermath of the violence but also the enduring consequences of trauma, loss and fear.^2^ The psychological ramifications of such a traumatic event extend beyond the immediate victims to encompass the entire community.^3,4^ Research indicates that such events can lead to widespread trauma, affecting not only direct victims but also their families, friends and the broader community,^5^ with shared experiences of violence potentially also impacting community dynamics, identity and resilience.^6^

Various governmental and non-governmental organisations mobilised resources to assist the affected community. Recognising the unique and unprecedented needs of the victims and the desire to provide culturally appropriate support, the Canterbury District Health Board (CDHB) recommended augmenting the existing mental health and social care workforce by recruiting Muslim professionals, particularly those with mental health experience, to work alongside established services.^7^ The critical role played by such professionals in facilitating recovery and resilience within traumatised communities has been reported in other contexts, highlighting in particular the importance of community trust and the potential role played by familiar individuals in the healing process.^8,9^ This approach was aligned with trauma-informed care principles, especially those emphasising cultural responsiveness, peer support and community trust. However, while these professionals were recruited to deliver trauma-informed care, many did not receive it themselves.

The urgency of the response and lack of infrastructure meant that their own psychological needs were often overlooked, despite their dual roles as both support workers and affected community members. As a result the roles created, while well intentioned, did not always align with best practices in trauma-informed care.^10^

## Trauma-informed care and professional challenges

Trauma-informed care is guided by principles such as safety, trustworthiness, peer support, collaboration, empowerment and cultural responsiveness.^11^ These principles aim to reduce re-traumatisation and promote healing in both clients and staff. In the Christchurch response, these principles were inconsistently applied.

Many people recruited for these positions were survivors or were close to victims, and some were placed in roles without adequate training, supervision or emotional support. Job descriptions were often ill-defined, and organisational expectations did not always take into account the emotional toll of working within one’s own traumatised community.

The authors of this study were directly involved in the post-attack response and bring both professional and lived experience to the research. While this insider perspective offers valuable insight, it also introduces potential biases, which are addressed through reflexive analysis and triangulation, as outlined in the section ‘Research team and reflexivity’, below.

Working in mental health, social care or community support positions is particularly challenging due to the psychological stressors inherent in these roles, which expose workers to the risk of vicarious traumatisation and compassion fatigue, occupational hazards that can significantly impair professional development and well-being.^12,13^ The cumulative impacts of burnout – emotional, mental and physical – can also impede the ability to provide effective services.^14^ Therefore, prioritising self-care is important for mitigating the stress and emotional reactions that may arise when working with clients.^15^

## Literature context and research gaps

Despite a growing recognition of these challenges, particularly within faith-based or other close-knit groups, the existing literature reveals a significant gap regarding the psychological consequences of navigating dual relationships in such contexts. Balancing personal grief with professional responsibilities can increase the risk of developing post-traumatic stress disorder (PTSD), vicarious traumatisation and secondary traumatic stress, especially when individuals are continually exposed to the trauma experienced by others.^16^ Recent studies reveal the necessity for tailored interventions addressing vicarious trauma that take into account the unique characteristics of diverse settings and demographics.^17^ The gap in empirical evidence highlights the need for further exploration of the experiences of professionals in different contexts, including in organisations led by migrants or refugees, which are increasingly common in Europe.^18^

While many professionals do not encounter substantial distress due to their work, a minority do exhibit clinically significant symptoms such as PTSD, which highlights the importance of targeted interventions to mitigate these impacts.^19^ Addressing these concerns is essential not only for improving their mental health but also for enhancing the quality of care provided to traumatised clients. Furthermore, there is also potential for growth and vicarious resilience to emerge in workers in post-traumatic contexts.^20–22^ A meta-synthesis of qualitative studies reveals that engagement in trauma-related work can significantly affect psychological well-being, leading to both adverse and beneficial transformations consistent with the frameworks of vicarious trauma and vicarious post-traumatic growth.^23^

The current research landscape is complicated by methodological challenges, including heterogeneity within study groups and a lack of comparison groups, which hinder the ability to draw firm conclusions about the psychological effects of trauma exposure. For this reason, there is a clear need for more scientifically rigorous investigations to understand the stressors faced by professionals in community settings, and to develop effective, evidence-based interventions. This study explores these dynamics within the context of workers impacted by the events of 15 March 2019.

The objective of this study was, therefore, to investigate, through qualitative analysis, the complexities of balancing dual roles within a close-knit community while managing personal grief and professional responsibilities. This investigation focused on two main issues: (a) the psychological and emotional challenges and (b) boundary challenges. Due to the large amount of data collected, this paper will primarily focus on the first issue, while a subsequent paper will report on boundary issues.

## Method

### Research design

This study employs a qualitative research design, grounded in a critical realist framework and with an ontological perspective that assumes that an individuals’ words offer insights into their unique version of reality. Qualitative research is inherently interpretive, aiming to explore the meanings and experiences associated with specific phenomena.^24^ Study reporting is based on the Standards for Reporting Qualitative Research (SRQR) checklist.^25^

### Research team and reflexivity

The research team comprises four Muslim academics researching the impacts of the 15 March 2019 mosque terrorist attacks. All are members of the Christchurch Muslim community and played significant roles in response and support initiatives following the attacks. Numerous issues described by participants had also concurrently been experienced by the researchers. Understanding these issues further was a motivation to conduct the study.

The team includes a senior research fellow with expertise in international health, who was a principal investigator for several studies related to the terrorist attack (R.S.-H.); a research fellow holding a PhD in Education (S.M.A.K.); an assistant research fellow and PhD candidate with a background in anthropology and qualitative research (F.A.); and a research assistant with a psychology background (R.L.A.). Three team members (S.M.A.K., F.A. and R.L.A.) also worked as interpreters and translators for the Bangladeshi, Pakistani and Arabic communities in Christchurch. The team were all involved in conducting interviews and data analysis.

The complexities and challenges associated with dual relationships in this research context are acknowledged. Recognising the potential influence of personal biases on data analysis and interpretation, the team used Avidnote software to triangulate results and offer an alternative analytical perspective for data examination. This additional layer of analysis provided a more neutral framework for data examination.

### Setting

Depending on participants’ preferences, interviews were conducted either in person at the Department of Psychological Medicine, University of Otago, Christchurch, at other appropriate locations or online via Zoom. All interviews were conducted by the research team, who are experienced in working with victims of the attacks, following trauma-informed ethical principles to ensure sensitivity and safety throughout the process.^11^ Mental health professionals from the department were available to support participants in the event of emotional distress.

### Participants

Participants were Muslim professionals working in government departments, non-government support agencies and academic institutions in Christchurch, who were recruited to work with the impacted community in a professional capacity following the 15 March 2019 attacks. People living outside Christchurch during the attacks, and those working in volunteer positions, were excluded.

Participants were selected using purposive sampling, based on their professional roles and involvement in the Christchurch Muslim community. As members of the same community, the team had direct access to the small number of potential participants and could approach them personally. This facilitated recruitment and built trust, helping to ensure that participants felt comfortable sharing their experiences.

Twenty participants were recruited from the identified occupational categories, a number considered adequate for achieving data saturation. The selection process was guided by an understanding of the community and the specific research context, with careful consideration given to balancing the roles and backgrounds of the participants. This approach, while not formally stratified, aimed to ensure comprehensive data collection while addressing practical constraints associated with in-depth analysis. The inclusion of participants from different demographics, such as varying age, gender and occupation, can enrich the inquiry, even if representativeness is not the primary goal. Overall, the sample size was designed to be practical and aligned with the research objectives, focusing on the richness rather than the quantity of the data.^24^

### Data collection

Semi-structured, one-to-one, qualitative interviews of 20–60 min were conducted in English between April and December 2023. An interview guide was developed to provide a comprehensive investigation of the research questions using a structured framework, with a series of open-ended questions that allowed for detailed and reflective responses (see Supplementary file 1 available at https://doi.org/10.1192/bjo.2025.10897).

Interviews were audio recorded and subsequently transcribed. To ensure the accuracy of the transcriptions, the research team meticulously cross-checked the transcripts against the original audio recordings. Following this verification process, participants had the opportunity to review their transcripts, to correct any inaccuracies, add supplementary information or clarify specific points; however, none of the participants took advantage of this. Identifying details were then removed, and participants were assigned anonymised identifiers, ranging sequentially from DR01 to DR20.

At the end of each interview, participants completed a checklist of common symptoms associated with burnout, compassion fatigue and vicarious traumatisation that they may have experienced as a consequence of their professional roles (see Supplementary file 2). They could select multiple symptoms as relevant. This approach allowed for the triangulation of qualitative and quantitative data. It is important to note that this checklist was not a standardised assessment tool; rather, it was compiled from existing literature reports.

### Data analysis

Interviews were analysed using reflexive thematic analysis, which emphasises critical reflection and aligns with the values of qualitative paradigms.^26^ Initially, the team comprehensively reviewed the data by listening to audio recordings and reading interview transcripts several times. Each person then independently generated initial codes. These were discussed and refined in an iterative process during team meetings, before being uploaded into NVivo 14 software for Windows (Lumivero, Denver, Colorado, USA; https://lumivero.com/products/nvivo/) for data management. The final themes were then defined and summarised to incorporate the principal findings.

Themes were then compared with those generated by Avidnote AI, software specifically developed for research applications, with advanced data security features that adhere to international standards (2024 for Windows; AvidemicAB, Goteborgs Stad, Sweden; https://avidnote.com). This can provide rapid analysis of individual transcripts, identify recurring themes and extract relevant quotations depending on the prompts used. It was used to corroborate findings from the manual analysis and provide supplementary insights, functioning similarly to an independent team member. Any insights produced by the software were subsequently discussed and evaluated against the original transcripts during team meetings.

The data were examined through a critical realist lens, to gain an understanding of how individual experiences may be shaped by broader social structures.

Several strategies were used to ensure trustworthiness, including member checking, which involved presenting the findings to participants to affirm the accuracy and credibility of the interpretations, prolonged engagement within the research context as well as an iterative review and discussion process during team meetings.^27^

## Results

Of the 20 participants recruited, 8 were men and 12 women, who worked for 9 different organisations. Their personal details are not reported, and their professional positions have been summarised into broad categories to preserve anonymity. Employment included government agencies (four people), research roles (seven people), community support services (eight people) and advisory or consultancy roles (four people). Some participants were employed in more than one role: this mostly included those who worked as interpreters (community support) in addition to their main job. The majority of participants were aged 30–50 years, with diverse ethnic backgrounds. All were members of the Christchurch Muslim community and who were living in the city at the time of the 2019 attack. Fourteen interviews were conducted in person and six using Zoom. Four major themes and several subthemes were identified, describing how these individuals navigated their dual roles working in the community, and the implications of such dual relationships on their personal and professional well-being.

### Theme 1: emotional and psychological toll

Participants were not only working as professionals, they were also victims of the attack and embodied tensions between individual agency and structural factors. This exacerbated the emotional and psychological burden experienced while working in a traumatic environment. A number of participants expressed a deep personal connection to their work, reinforcing their emotional vulnerabilities. There was an overwhelming sense of emotional burden and fatigue related to the impacts of the attack, and a sense that the work was never ending:

‘I have to deal with myself and as a person … I am a professional and it is really tough switching from one personal agenda to another. Sometimes I feel really down, emotionally and mentally, [and feel] that I should not continue this work’ (DR02).

‘I can’t talk about the impact it [the attack] has had on me, the way I miss them, or think about them at work because it is so personal’ (DR15).

‘It’s really hard because there is a job that does not finish’ (DR19).

The collective impact of the attack on the Muslim community was also emphasised:

‘We have this constant traumatisation going on for us as well. But you have also got to face it from some of the victim groups. I have been pushing every opportunity to say that the whole community has been impacted by this’ (DR14).

#### Vicarious trauma

Several participants experienced secondary traumatic stress because of their clients’ narratives, and this led to emotional exhaustion:

‘I was mentally affected because everyone is affected in the Muslim community … I was mentally affected listening to their stories’ (DR08).

‘When you have a long day of big job … you are struggling, and you feel so much pain’ (DR19).

These experiences can perpetuate a state of mental unrest, impacting both individual mental health and professional efficacy, as described by another participant:

‘Because I had around 30 to 35 clients … most of them were directly affected. [They] share the same story every time they need to visit or see an MP [Member of Parliament], or government official. So, they were repeating their stories … and I was facilitating all these things [and hearing multiple distressing narratives over and over again]’ (DR08).

Participants described being forced into a position of constant confrontation of grief and suffering, amplifying their own emotional burden:

‘When you are struggling with your own issues and injuries and you have to hear these things, while you are working for the community, its mentally very emotionally challenging’ (DR17).

#### Stress and burnout

Significant stress and burnout were also frequently reported, along with a lack of organisational support:

‘I was really, really burning out at this point. At the end I was at my max’ (DR01).

‘I am going home, and my cup is empty every night. I fall asleep crying’ (DR18).

‘In the beginning the support was very minimal. Because it was an evolving role. Nobody could understand what we go through. Because we were in a [non-Muslim] organisation. The fact was that we were just as badly traumatised as everybody else’ (DR06).

The lack of emotional support noted by some participants suggests that some organisations may not be adequately equipped to understand or address the psychological well-being of their employees. This can create an atmosphere where employees feel they must bear the weight of their work alone, leading to higher rates of burnout and attrition.

### Theme 2: the power of faith – altruism as a spiritual obligation

This theme highlighted a profound ethical and moral commitment ingrained in personal experiences and religious belief systems, which provided a framework for meaning-making and purpose that was shaped by cultural narratives and community norms.

#### Cultural and religious influence

Participants indicated that their actions were not just choices but rather responses to a perceived duty, informed by community expectations and internalised spiritual teachings:

‘I did it for the sake of God, not because I want to get a word from them … or get paid, I was going the extra mile to do things for the sake of my religion. Be a Muslim … give back to the community’ (DR19).

The interweaving of personal narratives with altruistic actions suggests that social and psychological components play significant roles in shaping their motivations to work in this space. Such insights can help inform community programmes aiming for social support, because understanding these motivations can enhance outreach and engagement effectiveness:

‘Even if I am feeling low, as a Muslim we believe that is our duty to look out for others and … that pushes and fuels me as a person … No, I can’t sit at home and pretend that no one else has a bigger problem than me. So, that helps me to overcome my selfish, pity kind of vision and step up’ (DR06).

‘Personally, I always feel better if I can do something for some people. If I can do some good for them, I feel satisfied’ (DR02).

This suggests that altruism is not merely an obligation for these individuals, but rather a source of personal satisfaction and purpose:

‘I felt it really helped me a lot, I think if I just sit at home and not helping them, I would feel regret’ (DR01).

#### Existential motivation

Many participants cited their Islamic beliefs as a significant motivator for their actions. The emphasis on giving back, serving others and fulfilling a divine duty reflects a strong connection between spirituality and altruism:

‘Because we are alive, and … because my husband is alive and I am one of the lucky people who got him back, I must make sure that those who were not as lucky as me, [that] they get my help’ (DR06).

‘I feel that, you know, none of my family were there that day … and that is part of the bigger plan, Allah’s bigger plan … there is a reason for that. So, I should help’ (DR14).

The existential belief that everyone has a role, coupled with the notion of being the ‘right person at the right time’, reflects a deeper understanding of purpose that may direct individual actions:

‘I can do it because I am the right person, with the right skills, in the right place, at the right time … and so I can do it, and therefore, I should. Because there is the belief that you are only given what you can handle’ (DR13).

### Theme 3: identity and perception – struggle for validation and belonging

The third theme involves social structures and identity, with the dissonance between public image and personal and professional realities being highlighted.

#### Self-perception

The experience of alienation may arise from various influences, including cultural, societal and interpersonal factors:

‘People have a different image of [me] … people who know me as interpreter or [in this role] … they did not see me as a very serious and responsible person [before]’ (DR03).

‘I do feel very out of place that I don’t kind of belong anywhere … I felt like I was a recent addition and … like a bit of an imposter’ (DR15).

#### Fractured identities and agency

Many participants expressed a fragmented sense of identity as they navigated their roles – suggesting not only internal struggles but also external pressures that dictated how they were viewed within the community:

‘At one point, I felt oh my God, in my whole life whatever image I have built around myself, I think it is going to be spoiled’ (DR17).

‘I really struggled with it because I felt I was brought into this role because of my connections, but then I can’t really perform because of my connections’ (DR15).

#### Trauma and social validation

The desire for validation of personal experiences, particularly in the context of trauma, also emerged as an important theme in this discourse:

‘Because we are not “victims” ourselves [i.e. not included in the official victim list], people don’t take us seriously and they don’t take our traumas and our experiences seriously’ (DR14).

Another notable concern was the criticism and condemnation that some individuals encountered from their community:

‘People coming up and they say, to your face, why are you doing this? You know in a nasty way, why you involved? Like you are benefitting in some way’ (DR14).

‘There is always going to be condemnation and criticism’ (DR13).

These quotations reflect a critical intersection of identity, belonging and societal perception. They reveal narratives marked by contradiction: the desire to engage authentically while grappling with external perceptions that diminish their contributions and experiences. Participants’ descriptions of their own trauma and lack of acknowledgement from others underscore a critical realist view of the importance of social validation; they reveal the psychological burden imposed by societal attitudes and the need to validate diverse experiences of trauma. This lack of recognition may perpetuate feelings of alienation, hindering their own healing process.

### Theme 4: support systems – limitations and resource needs

The final theme revealed significant gaps in formal support structures.

#### Structural limitations

In some professional contexts, the provision of support was perceived to be insufficient. This was particularly troubling for some interpreters who were confronted by graphic details of the attacks at work, yet were given no opportunity for debriefing or support following their work sessions. Consequently, they reported returning home while still bearing the psychological weight of the distressing information they had encountered:

‘On top of that, again and again it [working as an interpreter] introduces me to the traumatic situations … it reminds me that these things happened’ (DR02).

‘There was no support there, not at all. It was the most tough job … whenever I went to court [as an interpreter], I always came back sad … It was affecting my mental health … but never did I [even] get an email saying how are you feeling’ (DR03).

‘There was very little realisation, particularly in those early days, of the extent to which talking about the terrorist by name, talking about the terrorist in terms of what he did or didn’t do, or what he was thinking [was impacting me]’ (DR13).

#### Informal support and collective agency

Further critical areas relating to the need for better support frameworks within work environments involving traumatic contexts were revealed:

‘In the beginning, the support was very minimal … we just had ourselves, the team, we didn’t even have a team leader’ (DR6).

Several participants emphasised the importance of informal peer support from their Muslim colleagues who had shared experiences. The nature and emotional intensity of the work meant that conversations with trusted colleagues became an outlet for catharsis:

‘I think most of my support at work, probably comes through the team … [at] some of our meetings, we can talk about things … because [they understand and] we have got that collective confidentiality’ (DR14).

Several participants expressed a preference for informal discussions and open dialogues in collaborative team settings. Such informal conversations, initiated by employees, lack the protocols and pressures of formal supervision sessions. They reported that this contributed to the establishment of meaningful interpersonal connections, providing a valuable source of emotional relief:

‘These informal discussions are actually very much [more] helpful than any formal supervision’ (DR17).

‘I get a lot more support from those shared experiences and just talking to my colleagues, rather than formal supervision sessions’ (DR14).

#### Cultural competence

Some participants reiterated the need for culturally competent leadership and supervision. Having leaders or supervisors who can relate to, or understand, their experiences significantly improved their overall support and well-being:

‘A lot of the time with supervision, if it’s not a Muslim supervisor … you have to explain it to them. You have to spend time out of your one-hour supervision to try to explain it to them and even then, maybe they will get it or maybe they won’t. But with your team, it’s the Muslim team … they get it’ (DR15).

‘When they appointed a Muslim community representative [it was better] because we know our community better than anyone’ (DR17).

#### Common symptoms

**Fig. 1:**
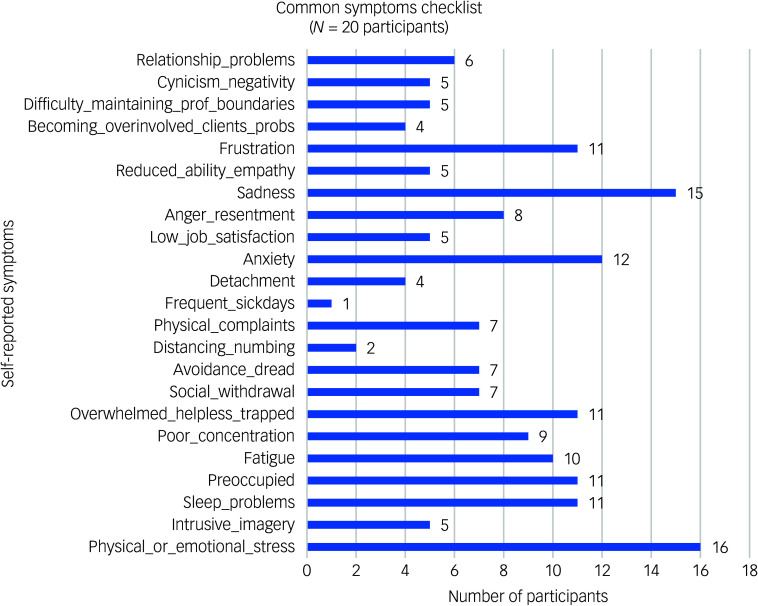
Frequency of self-reported common physical and psychological symptoms.

Physical or psychological symptoms reported as a result of their work are shown in Fig. 1.

Stress was common, being reported by 80% of participants. The majority of people also reported feeling sad (75%) or anxious (60%) due to their jobs. Over half of those interviewed (55%) indicated that they felt frustrated, overwhelmed, helpless or trapped in their jobs. Others were preoccupied with thoughts of their clients or had trouble sleeping. Taking time off work was rare, with only one person reporting that they frequently did this.

## Discussion

This study examines emotional and psychological challenges faced by Muslim staff in the aftermath of the 15 March 2019 terrorist attacks on two mosques in New Zealand. The findings reveal a complex interplay between individual agency and structural factors that shape the lived experiences of these individuals. Participants described a landscape marked by significant emotional burdens, impacted by the dual roles they occupy – those of professional support workers and community members affected by the traumatic events.

The first major theme, emotional and psychological toll, reveals the multifaceted nature of stress experienced by participants, reported in the interviews and in the symptom checklist, which indicated high rates of stress, sadness and anxiety. The findings suggest that the emotional challenges are not necessarily individual but may also stem from systemic issues related to the organisations in which they work. For instance, participants reported feeling overwhelmed and emotionally fatigued, suggesting a disconnect between organisational expectations and the individual capacity to process trauma. This aligns with existing literature highlighting the effects of vicarious trauma and emotional exhaustion on professional efficacy.^14,28,29^

The data point to a need to formally acknowledge and address the psychological well-being of employees. The challenge of balancing dual roles, as both members of the traumatised community and professionals, can result in considerable emotional strain. The acknowledgment of unresolved trauma highlights the need for sustained psychological support for affected individuals.^9,10^ While participants in some organisations reported significant emotional challenges and a lack of support, it is important to note that staff within established agencies, including government departments, typically had access to sufficient training and resources such as counselling and professional supervision, that helped them to manage their trauma. However, even in these situations, the resources may not have been culturally appropriate, limiting their effectiveness in addressing the unique needs of diverse employees. By contrast, those whose roles were created in response to the attacks appeared to face greater challenges, indicating a disparity in support structures. This highlights the potential issues associated with roles that are established rapidly in response to crises such as disasters and refugee emergencies. These findings also suggest that, while recruitment efforts were guided by trauma-informed care principles such as cultural responsiveness and peer support, these principles were not consistently extended to the professionals themselves. The lack of emotional assistance noted by some participants in these newly established positions reveals systemic inadequacies that exacerbate feelings of isolation and burnout. Simultaneously, this disconnection may manifest in high attrition rates and decreased job satisfaction, underscoring the importance of integrated mental health resources, peer support groups and training to equip employees for the emotional demands of their roles. Organisations need to prioritise mental health frameworks that facilitate both personal recovery and professional sustainability.

Another notable finding is the role of altruism, which was identified as a significant motivating force closely associated with participants’ faith and cultural identity. Framed within a narrative of moral obligation, many participants revealed that their religious beliefs provided a sense of purpose and fulfilment in their work. They indicated that their commitment to helping others not only benefitted their communities but also helped them cope with their own grief and trauma. Research has highlighted the importance of altruism and empathy in contributing to the psychological well-being of healthcare professionals. This aligns with existing theories that propose a relationship between altruistic behaviour and improved emotional well-being.^30^ It can significantly mitigate occupational stress and burnout, while concurrently promoting a sense of purpose and enhancing social connectedness among individuals.^31^ Furthermore, a positive correlation has been established between emotional empathy and altruism, with empathy being recognised as a key determinant of altruistic behaviour within the healthcare workforce.^32^

In the aftermath of terrorist attacks, the experiences of professionals are often influenced by their relationships with colleagues. Supportive interactions can mitigate feelings of anxiety and social isolation.^33^ For instance, mental health professionals involved in post-disaster care, such as those responding to the 11 September 2001 attacks in the USA, initially experienced high levels of stress. However, they reported long-term positive impacts on their personal lives, describing their work as ultimately rewarding despite the emotional challenges presented by their clients’ experiences.^34^ This dynamic is also relevant when considering the role of peers in refugee contexts, particularly in regard to providing mental health support.^35^ Although such interventions are more effective when culturally responsive and tailored to the needs of diverse populations, there are gaps in understanding the challenges faced by former refugees in these roles.

The theme of identity and perception illuminates participants’ struggles for validation and belonging, particularly in the wake of traumatic experiences. Participants encountered societal scrutiny that undermined their professional contributions, reflecting broader discourses on marginalisation and identity politics within minority communities.^36^ Such narratives point to the critical intersection between personal trauma and public perception, revealing that a lack of acknowledgment from clients and inter-group tensions can exacerbate stresses and feelings of exclusion, potentially hindering the healing process and compounding emotional distress for workers. Support frameworks that encourage validation of diverse experiences, and promote an inclusive environment where contributions can be recognised, are essential.

The study revealed significant gaps in formal support systems for front-line workers, particularly interpreters. There were limited debriefing opportunities and appropriate psychological support, which not only further exacerbated the emotional burden they faced but also highlighted an urgent need for tailored mental health resources. The relevance of informal support mechanisms emerged as another important theme, in particular the emphasis on the positive benefits of shared experiences and peer support, aligning with existing literature.^37^ The importance of culturally competent supervision was also mentioned. Participants noted that supervisors who understand their cultural contexts are better equipped to facilitate discussions about trauma and its effects, resulting in better relationships and improved therapeutic outcomes.

Organisations should, therefore, implement strategies to support both institutional and informal support systems. In particular, supervisors should be trained to recognise the unique challenges faced by staff and provide tailored support that acknowledges the cultural dimensions of trauma. In addition, they should monitor the level of exposure experienced by front-line workers, and understand that the demands of their roles should not be equated with those in non-triggering environments. This may include the necessity for reduced hours and workload expectations to prevent burnout. For professionals navigating dual roles, it is essential to provide culturally competent support that acknowledges their complex experiences. Informal opportunities for peer support should be encouraged, because these interactions are important for validation and shared understanding. It is particularly important to facilitate such opportunities for those in isolated roles.

Finally, the role of faith and altruism in the lives of these professionals warrants further investigation. While these elements may enhance resilience and promote post-traumatic growth, they could potentially also encourage individuals into work that jeopardises their psychological well-being.

### Limitations and future research

Several limitations in this research must be acknowledged. First, the shared community background of the researchers could introduce potential biases. Additionally, the small sample size of 20 participants limits the extent to which findings can be generalised to other populations. Individuals not included in this sample may have experienced different psychological impacts that are not captured in this study. In addition, utilising a standard measurement tool would provide more accurate insights into participants’ mental health status.

The insights from this study provide a valuable framework to improve trauma-informed approaches for disaster response initiatives. They provide unique perspectives on the interconnectedness of faith, identity and support mechanisms in the workplace. While this investigation centres on a specific incident, the results have broader significance, especially regarding peer support initiatives for refugees and asylum seekers in Western countries. Future research could further examine the complex experiences of professionals within other small, distinctive communities, particularly those who navigate dual roles in the context of trauma. Such investigations are essential to develop comprehensive strategies that effectively address the unique needs and challenges of employees in these critical positions.

## Supporting information

Sulaiman-Hill et al. supplementary material 1 Sulaiman-Hill et al. supplementary material

Sulaiman-Hill et al. supplementary material 2 Sulaiman-Hill et al. supplementary material

## Data Availability

The data from the qualitative interviews are not publicly available because they contain material that could compromise the privacy of research participants.

## Supplementary material

The supplementary material is available online at https://doi.org/10.1192/bjo.2025.10897
