# Supplementary material for: Navigating dual roles: qualitative exploration of the psychological impacts on Muslim professionals supporting their community after a terror attack
Source: BJPsych Open. 2025 Nov 5;11(6):e264. doi: 10.1192/bjo.2025.10897 (PMC12641404; doi:10.1192/bjo.2025.10897)
Supplement: Sulaiman-Hill et al. supplementary material 1 — Sulaiman-Hill et al. supplementary material [file S2056472425108971sup001.docx]

**Table 1. Question prompts for semi-structured interviews**

| **Semi-structured Question prompts** |
| --- |
| 1. Can you tell me about your involvement in the Christchurch Muslim community before and after the attacks, i.e., before you started your professional role? |
| 2. Can you explain your role(s) working with the Muslim community after the March 15 attacks? |
| 3. What have been some advantages of using Muslim staff for your role(s)? Generally, and for you personally? |
| 4. How do you deal with seeing clients at social events? |
| 5. Has your professional role affected your relationships in the community? |
| 6. How well do you think you have been supported in your role to deal with these dual relationship challenges? E.g., Did you receive supervision as part of your job? |
