# Supplementary material for: Navigating dual roles: qualitative exploration of the psychological impacts on Muslim professionals supporting their community after a terror attack
Source: BJPsych Open. 2025 Nov 5;11(6):e264. doi: 10.1192/bjo.2025.10897 (PMC12641404; doi:10.1192/bjo.2025.10897)
Supplement: Sulaiman-Hill et al. supplementary material 2 — Sulaiman-Hill et al. supplementary material [file S2056472425108971sup002.docx]

**Table 2: Common symptoms of Vicarious Trauma, Burnout & Compassion fatigue checklist**

| **Tick** | **Possible symptoms related to work**  **Please tick all of the issues below that you have experienced as a result of your job** |
| --- | --- |
|  | Physical or emotional stress |
|  | Intrusive imagery |
|  | Sleep problems |
|  | Pre-occupied with thoughts of your client outside of work |
|  | Fatigue |
|  | Poor concentration |
|  | Feeling overwhelmed, helpless or trapped in your job |
|  | Social withdrawal |
|  | Avoidance or dread of meeting with certain clients |
|  | Distancing or numbing |
|  | Physical complaints e.g. headache, muscle tension, cardiac symptoms |
|  | Frequent sick days |
|  | Detachment |
|  | Anxiety |
|  | Low job satisfaction |
|  | Anger & resentment |
|  | Sadness |
|  | Reduced ability to feel empathy |
|  | Frustration |
|  | Becoming overly involved with your client’s problems – difficulty in maintaining professional boundaries |
|  | Cynicism, negativity or feeling judgemental towards clients |
|  | Relationship problems |
